# Ultrasound-Guided Microwave Ablation for Benign Thyroid Nodules in Older Adults: Comparative Analysis of Clinical Outcomes and Safety

**DOI:** 10.3390/diagnostics16172866

**Published:** 2026-09-07

**Authors:** Feyza Mutlay, Mehmet Yuksek, Saim Turkoglu, Zehra Kara

**Affiliations:** 1 Division of Geriatrics, Department of Internal Medicine, Faculty of Medicine, Canakkale Onsekiz Mart University, Canakkale 17000, Türkiye; 2 Department of Radiology, Van Training and Research Hospital, University of Health Sciences, Van 65170, Türkiye; mehmetyuksek65@yandex.com; 3 Department of Radiology, Faculty of Medicine, Yuzuncu Yıl University, Van 65090, Türkiye; mdsaimturkoglu@gmail.com; 4 Department of Endocrinology and Metabolism Diseases, Memorial Şişli Hospital, Istanbul 34360, Türkiye; drzehrakara@yahoo.com

**Keywords:** microwave ablation, thyroid nodules, older adults, minimally invasive treatment, volume reduction rate

## Abstract

**Background:** The management of benign thyroid nodules causing compressive symptoms, cosmetic concerns, or rapid growth in older adults is challenging because of multimorbidity and increased surgical risk. Ultrasound-guided microwave ablation (MWA) is a minimally invasive treatment option; however, data regarding its efficacy and safety in older patients remain limited. This study compared clinical outcomes of MWA between older and younger adults with benign thyroid nodules. **Methods:** This retrospective comparative study included 98 patients with benign thyroid nodules causing compressive symptoms, cosmetic concerns, or rapid growth (defined as more than a twofold increase in volume within 6 months) who underwent ultrasound-guided MWA. Patients were divided into older adults (≥60 years, *n* = 33) and younger adults (18–59 years, *n* = 65). Nodule volume, volume reduction rate (VRR), symptom and cosmetic scores, thyroid function, and procedure-related complications were evaluated at baseline and 3- and 6-month follow-up. Comorbidity burden was assessed using the age-adjusted Charlson Comorbidity Index (aCCI). **Results:** MWA significantly reduced nodule volume in both groups. At 6 months, median VRR was comparable between older and younger patients (66.1% vs. 66.7%, *p* = 0.50). Symptom and cosmetic scores significantly improved in both groups (for both groups *p* < 0.001), while thyroid function remained stable. Despite higher comorbidity burden among older patients, treatment outcomes were similar between groups. No major complications occurred; transient hoarseness developed in two younger patients and resolved during follow-up. **Conclusions:** Ultrasound-guided MWA appears to be an effective and safe minimally invasive treatment for benign thyroid nodules in older adults. Comparable outcomes between older and younger patients suggest that age and comorbidity burden should be considered in clinical decision-making rather than as absolute limitations to treatment.

## 1. Introduction

Thyroid nodules are among the most common endocrine disorders and are being detected with increasing frequency because of the widespread use of high-resolution imaging modalities. Their prevalence increases substantially with age. A meta-analysis demonstrated that thyroid incidentalomas are identified in approximately 42% of individuals older than 50–55 years compared with 20% of those younger than 50–55 years [1]. Consistent with these findings, a large ultrasound-based cross-sectional study using electronic health records reported that thyroid nodule prevalence increases progressively with age and is influenced by several demographic and clinical factors [2]. Similarly, a population-based epidemiological study from China reported prevalence rates of 32.23% among individuals aged 60–69 years and 36.01% among those aged ≥70 years [3]. As global life expectancy continues to rise, the evaluation and management of thyroid nodules in older patients has become an increasingly important clinical challenge.

The diagnostic evaluation of thyroid nodules generally follows the same principles in older and younger patients. Nevertheless, management decisions in older patients often require individualized assessment because of age-related comorbidities, increased surgical risk, and differences in life expectancy. Despite these considerations, evidence supporting age-specific treatment strategies for thyroid nodules remains limited.

Minimally invasive thermal ablation (TA) techniques have emerged as effective alternatives to surgery for the treatment of symptomatic benign thyroid nodules [4]. These approaches are particularly valuable for older patients with significant comorbidities, those at increased operative risk, or those who decline surgical intervention [5]. Compared with thyroid surgery, TA is associated with lower complication rates while avoiding potential surgical sequelae such as recurrent laryngeal nerve injury, hypocalcemia, postoperative bleeding, cervical scarring, lifelong thyroid hormone replacement therapy, and deterioration in quality of life [6].

Among available TA modalities, laser ablation and radiofrequency ablation have been extensively investigated, whereas microwave ablation (MWA) has gained increasing attention because of its favorable technical characteristics. Unlike radiofrequency ablation, MWA generates electromagnetic energy without requiring an electrical circuit, making it less susceptible to heat-sink effects and potentially more suitable for patients with implanted electronic devices or certain cardiac comorbidities. Recent studies have demonstrated that MWA is a safe and effective treatment for benign thyroid nodules, producing substantial volume reduction with a low incidence of complications [7,8].

Although the efficacy and safety of MWA have been reported in several single-center and multicentre studies, evidence specifically focusing on older patients remains scarce. Furthermore, direct comparisons of treatment outcomes between older and younger patients using standardized treatment protocols are limited. Given the increasing number of older patients requiring treatment for symptomatic benign thyroid nodules, understanding whether age influences the effectiveness and safety of MWA has important clinical implications.

Therefore, this study aimed to compare the efficacy and safety of microwave ablation in older and younger adult patients with benign thyroid nodules by evaluating clinical outcomes, nodule volume reduction, thyroid function, and procedure-related complications.

## 2. Materials and Methods

### 2.1. Study Design and Patient Selection

This retrospective study was approved by the Scientific Ethics Committee of the University of Health Sciences, Van Training and Research Hospital (Approval No. 30303.8/25). The study was conducted in accordance with the principles of the Declaration of Helsinki.

Between September 2022 and June 2023, all patients with benign thyroid nodules who were at high surgical risk or declined surgery and therefore underwent ultrasound-guided microwave ablation (MWA) were retrospectively analysed [9]. Patients were stratified into two age groups: adults (18–59 years) and older patients (≥60 years). Although 65 years is commonly used as an age threshold in geriatric medicine, a cutoff of 60 years was selected in the present study based on previously published epidemiological and clinical studies [10,11]. The study population comprised 98 patients, including 65 adults and 33 older patients.

Demographic characteristics, clinical findings, and laboratory parameters, including serum thyroid-stimulating hormone (TSH), free triiodothyronine (fT3), and free thyroxine (fT4), were collected from electronic medical records. Comorbidity burden was assessed using the age-adjusted Charlson Comorbidity Index (aCCI) [12]. Symptom severity was evaluated using a 10-point visual analog scale (VAS), with higher scores indicating greater symptom burden. Cosmetic concerns were assessed using a physician-rated four-point cosmetic scoring system, ranging from 1 (no palpable mass) to 4 (readily visible cosmetic deformity) [13].

### 2.2. Inclusion Criteria 

Patients with Bethesda Category II thyroid nodules measuring ≥2 cm associated with compression symptoms (such as dysphagia, dyspnea, or hoarseness), cosmetic concerns, or continued growth during ultrasound follow-up were included. Patients with euthyroidism, hypothyroidism, or subclinical hyperthyroidism were also eligible [9].

### 2.3. Exclusion Criteria

Nodules with fine-needle aspiration biopsy results not classified as Bethesda Category 2; patients receiving radioactive iodine, antithyroid drugs, and iodine-containing medications; overt hyperthyroidism; pregnant or breastfeeding patients; patients with retrosternal goiter, contralateral vocal cord dysfunction, bleeding disorders, or severe cardiovascular insufficiency; and patients with incomplete clinical data were excluded from the study.

### 2.4. Biochemical Measurements

For biochemical measurements, peripheral venous blood samples were collected after an 8-h fast. In patients taking levothyroxine, blood was drawn before taking the drug that day. The hormone values for TSH, free T3, and free T4 were determined from the blood samples.

### 2.5. Ultrasound Evaluation, Fine-Needle Aspiration, and Microwave Ablation Procedure

All ultrasound (US) examinations, fine-needle aspiration (FNA) biopsies, and microwave ablation (MWA) procedures were performed by the same experienced operator to minimize interobserver variability. Baseline and follow-up assessments were conducted using a high-resolution ultrasound system equipped with an 11L-4 linear transducer and color Doppler imaging (Aplio 500, Toshiba Medical Systems, Otawara, Japan). Nodule characteristics, including composition, echogenicity, margins, calcifications, vascularity, and volume, were evaluated according to the American College of Radiology Thyroid Imaging Reporting and Data System (ACR TI-RADS) [14].

All symptomatic thyroid nodules classified as ACR TI-RADS categories 1–5 were evaluated for MWA. A symptomatic nodule was defined as one causing compressive symptoms, cosmetic concerns, or rapid growth, defined as more than a twofold increase in volume within 6 months. Patients with solitary or multiple symptomatic nodules measuring ≥2 cm were considered eligible for treatment. In patients with multinodular goiter, each nodule scheduled for ablation underwent independent cytological evaluation. Nodules classified as ACR TI-RADS 1 or 2 underwent a single US-guided FNA biopsy. In contrast, nodules classified as ACR TI-RADS 3–5 underwent two separate US-guided FNA biopsies to confirm benign cytology. Only nodules with benign cytopathological findings (Bethesda category II) were scheduled for MWA.

Before the procedure, thyroid function tests were reviewed, and anticoagulant or antiplatelet therapy was temporarily discontinued or adjusted according to current clinical recommendations to minimize the risk of bleeding. Immediately before ablation, all patients received intravenous paracetamol and weight-adjusted methylprednisolone (1 mg/kg) to reduce procedural pain, edema, and post-procedural inflammatory reactions.

MWA was performed under local anesthesia with continuous real-time US guidance. After local anesthetic infiltration, hydrodissection was performed by injecting 0.9% normal saline around the thyroid capsule to create a protective fluid barrier between the target nodule and adjacent critical structures, including the recurrent laryngeal nerve, trachea, esophagus, and carotid sheath. Saline injection was continued throughout the procedure whenever necessary to maintain an adequate safety margin.

**Figure 1:**
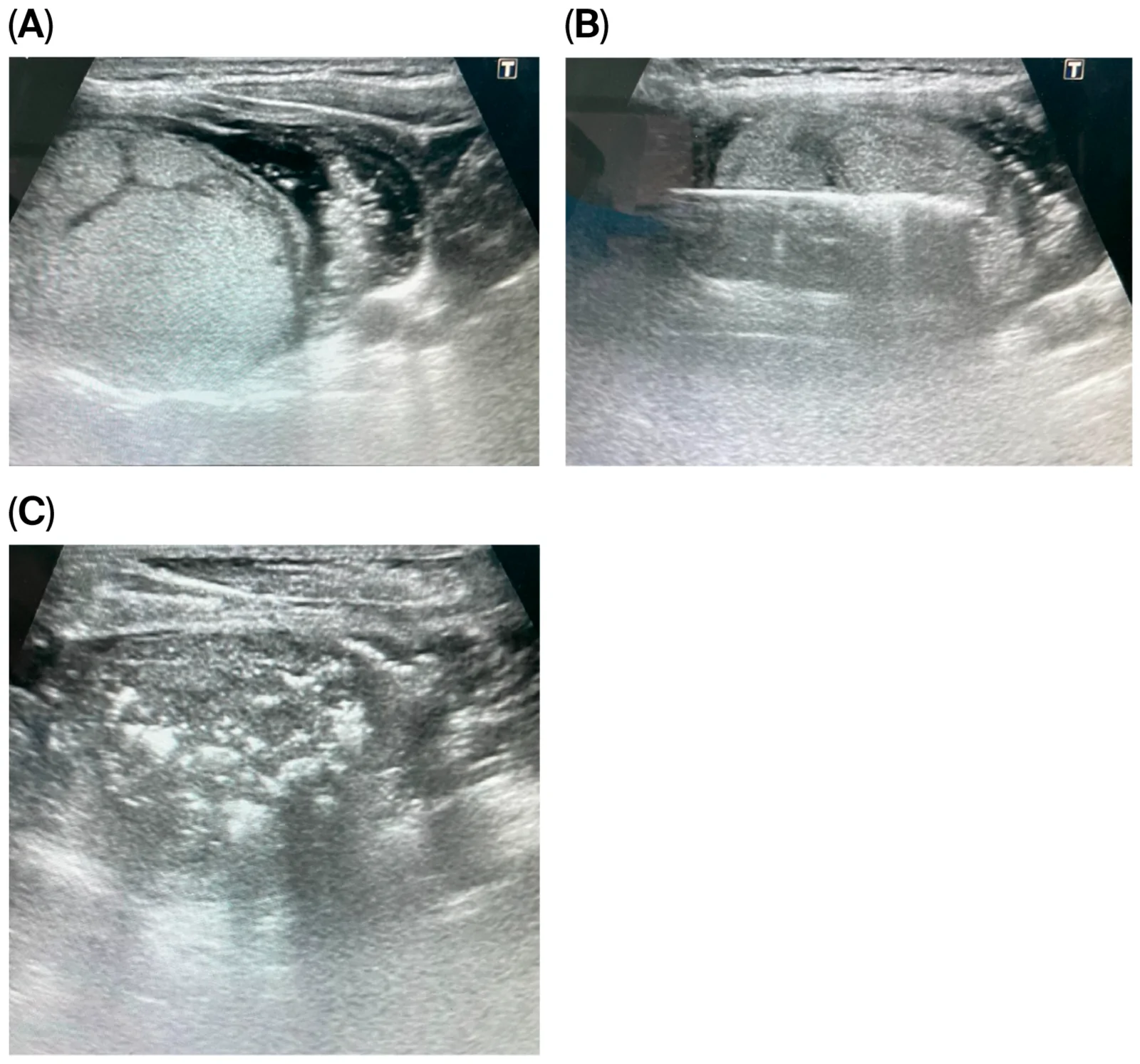
(**A**) Ultrasound-guided hydrodissection creating a protective fluid barrier between the thyroid nodule and adjacent critical structures. (**B**) Hyperechoic microbubbles generated during microwave ablation of the thyroid nodule. (**C**) Post-ablation ultrasound image demonstrating the ablation zone within the treated thyroid nodule.

The microwave antenna was inserted into the target nodule using a trans-isthmic approach whenever anatomically feasible. Ablation was initiated at 20 W using a pulsed energy delivery technique. Output power was gradually increased, when required, to a maximum of 40 W according to nodule size and the extent of the ablation zone, with a mean operating power of approximately 30 W. As hyperechoic microbubbles developed during thermal coagulation, the antenna was sequentially repositioned using the moving-shot technique to achieve complete and homogeneous coverage of the nodule while minimizing tissue carbonisation (Figure 1).

The endpoint of ablation was defined as complete disappearance of intranodular vascularity on color Doppler US and complete coverage of the targeted lesion by the transient hyperechoic ablation zone. Particular care was taken to preserve the thyroid capsule and avoid thermal injury to surrounding critical structures. Continuous verbal communication with the patient was maintained throughout the procedure to facilitate early detection of recurrent laryngeal nerve irritation or voice changes, and vocal cord motion was additionally monitored using real-time ultrasound.

All procedures were performed using the ECO-200G Microwave Ablation System (ECO Medical Technology Co., Ltd., Nanjing, China) equipped with a 16-gauge antenna (10 cm shaft length, 3 mm active tip). The procedure duration ranged from 7 to 30 min (mean, 17 min), depending on nodule size and procedural complexity. Following treatment, patients were observed for approximately 1 h and discharged after confirmation of stable vital signs and the absence of immediate procedure-related complications.

### 2.6. Statistical Analysis

Statistical analyses were conducted using the Statistical Package for the Social Sciences (SPSS) software (version 21.0). Data were assessed for normality using the Kolmogorov–Smirnov test. Continuous variables were reported as mean ± standard deviation (SD) and/or median (interquartile range [IQR]). For normally distributed data, Student’s *t*-test was used to compare groups. Medians were compared using the Mann–Whitney U test and the Kruskal–Wallis test. Correlations between variables were assessed using Spearman and Pearson tests, based on the data distribution. The Wilcoxon Signed-Rank Test was used to compare two dependent variables. To analyze non-normally distributed data, the non-normally distributed module of the One-Way ANOVA test (Tamhane’s T2) was employed. Results were examined using a 95% confidence interval, and a *p* value of less than 0.05 was considered statistically significant.

### 2.7. Sample Size

The required sample size was calculated using the G*Power program (version 3.1.9.7). We found that the total sample size required to test the difference between two dependent means at a 5% significance level, 85% power, and an effect size of 0.5 was 31. The total sample size required to test the difference between two independent means at a 5% significance level, 80% power, and an effect size of 0.6 was found to be 72. The total sample size required to test the difference between three independent means, with a 5% significance level, 95% power, and an effect size of 0.25, was found to be 36. The total sample size required to test the difference between three independent means, with a 5% significance level, 80% power, and an effect size of 0.4, was found to be 66. The total sample size required to test the correlation between two different variables, with a 5% significance level, 80% power, and an effect size of 0.4, was found to be 44. The total sample size required to test the multivariate regression analysis with a 5% significance level, 80% power, and an effect size of 0.15 was found to be 68.

## 3. Results

A total of 98 patients, consisting of 23 men (23%) and 75 women (77%), were included in our study. Thirty-three (33%) were older patients, and 65 (67%) were adult patients. The mean ages were 68.4 ± 6.9 years for the older group and 43.7 ± 8.1 years for the adult group. The demographic, clinical, and laboratory characteristics of the patients are listed in Table 1.

### 3.1. Clinical Findings

In the older group, the symptom score was 8 [7,8] before MWA and 4 [3,4] after 6 months (*p* < 0.001, effect size = 0.5); the cosmetic score was 2 [2,3] before MWA and 1 [1,2] after 6 months (*p* < 0.001, effect size = 0.5). In the adult group, the symptom score was 7 [7,8] before MWA and 3 [2–4] after 6 months (*p* < 0.001, effect size = 0.5); the cosmetic score was 3 [2,3] before MWA and 1 [1,2] after 6 months (*p* < 0.001, effect size = 0.5).

In the adult group, temporary hoarseness occurred as a complication after MWA in two patients with nodules measuring 13 and 24 mL, respectively. Both patients were started on methylprednisolone at a dose of 16 mg twice daily [15,16], and their vocal cords were monitored with ultrasound. Hoarseness resolved after 1 and 3 months, respectively.

### 3.2. Endocrinological Results 

Before MWA, 7 (7.1%) of the patients had hypothyroidism and were taking levothyroxine, 73 (74.4%) were euthyroid, and 18 (18.5%) had hyperthyroidism. Isoactive nodules were present on the thyroid scintigraphy scans of patients with hyperthyroidism. None of the patients with subclinical hyperthyroidism were taking antithyroid medication; two patients were taking beta-blockers. No change in thyroid function was detected after the MWA procedure. The functional status of the thyroid gland in the groups is shown in Table 2.

### 3.3. Radiologic Results

The structural characteristics of thyroid nodules are shown in Table 2. The mean nodule volume was 11.2 mL (6.1–21.4 mL) (Md [IQR]). Among the nodules, 45 (45.9%) were solid, 12 (12.2%) were cystic, and 41 (41.8%) were mixed. Additionally, 41 (41.8%) nodules were hypoechoic, 39 (39.7%) were isoechoic, and 18 (18.3%) were hyperechoic. Vascularization was observed in 97 nodules and was not detected in 1 nodule.

The nodule volumes of the groups in the post-up examinations after MWA are listed in Table 3. After MWA, the volume regression rate at month 3 was 48.6 [35.7–59.7]% in the older group and 52.9 [36.8–63.4]% in adults (*p* = 0.7, CI 95%, −0.07_0.09, effect size = 0.6). The volume regression rates at month 6 were 66.1 [55.3–77.5]% for the older group and 66.7 [55.3–74.1]% for adults, respectively (*p* = 0.5, CI 95%, −0.08_0.04, effect size = 0.6).

In the older group, the rate of volume reduction did not vary according to nodule structure: solid: 61.2% [54–70], cystic: 77% [51–90], and mixed: 68% [56–77] (*p* = 0.4, effect size = 0.4). In the adult group, the rates were: solid: 67.4% [53.4–76.2], cystic: 54.7% [43.5–73.7], and mixed: 67% [60–71.3] (*p* = 0.37, effect size = 0.4).

The aCCI score was 2 [0–3] for solid nodules, 2 [1–3] for cystic nodules, and 3 [1–7] for mixed nodules (*p* = 0.001, effect size = 0.4).

### 3.4. Correlation Analysis

When the correlation between nodule volume shrinkage and nodule structure was examined, mixed nodules showed greater shrinkage in nodule volume (*p* = 0.032, r = 0.219, CI 95% 0.021–0.4, effect size = 0.4). A weak association was found between age and nodule structure; the proportion of mixed nodules increased with age (*p* = 0.03, r = 0.22, CI 95% 0.022–0.4, effect size = 0.4). However, a strong correlation was found between aCCI and nodule structure; the frequency of mixed nodules increased with aCCI (*p* = 0.001, r = 0.347, CI 95% 0.16–0.51, effect size = 0.4). In the relationship between functional status and nodule structure, the mixed nature of the nodule and its hyperthyroid characteristics were strongly and positively correlated (*p* < 0.001, r = 0.395, CI 95% 0.21–0.55, effect size = 0.4). A similarly strong association was found when aCCI was compared with thyroid functional status; hyperthyroidism was correlated in the same direction with aCCI (*p* = 0.002, r = 0.324, CI 95% 0.13–0.49, effect size = 0.4).

### 3.5. Regression Analysis

In the subgroup analysis stratified by sex, we performed regression analysis of the MWA-induced VRR, against nodule structure, functional status, echogenicity, and aCCI. In men, a relationship was found between the functional characteristics of the thyroid nodule and the VRR (R^2^ = 0.496, β = 0.094, *p* = 0.043, confidence interval: 95%: 0.003–0.184, effect size = 0.15). In women, however, no relationship was observed between the nodule shrinkage rate and any of the four independent variables.

In the multicollinearity test, no multicollinearity was detected among nodule structure, functional status, echogenicity, and aCCI (VIF values: 1.23, 1.04, 1.24, and 1.23, respectively).

## 4. Discussion

The present study demonstrates that ultrasound-guided microwave ablation (MWA) is an effective and safe treatment for symptomatic benign thyroid nodules in both older and younger adults. The principal findings were that (i) MWA produced substantial reductions in nodule volume together with significant improvements in symptom and cosmetic scores; (ii) treatment efficacy was comparable between older and younger patients despite the substantially higher comorbidity burden in the older cohort; (iii) thyroid function remained stable throughout follow-up; and (iv) procedure-related complications were uncommon and limited to transient hoarseness in two younger patients. These findings further support previous international recommendations that thermal ablation should be considered an effective minimally invasive alternative to surgery for carefully selected patients with benign thyroid nodules [4,5,17,18]. 

Management of benign thyroid nodules in older patients requires balancing treatment efficacy against procedural risk. Older patients frequently present with multiple chronic diseases, frailty, polypharmacy, and reduced physiological reserve, all of which may increase the risk of surgery and postoperative complications. Consequently, international guidelines increasingly advocate minimally invasive thermal ablation techniques for symptomatic benign thyroid nodules in patients who are poor surgical candidates or prefer to avoid surgery [19,20]. Our findings extend these recommendations by demonstrating that advanced age itself does not appear to compromise the therapeutic response to MWA. Although the older cohort had significantly higher age-adjusted Charlson Comorbidity Index (aCCI) scores, their volume reduction rates and clinical improvements were comparable to those observed in younger adults, suggesting that chronological age alone should not preclude consideration of MWA. 

However, the present findings should be interpreted in the context of our predefined inclusion criteria. Only benign thyroid nodules ≥ 2 cm with compressive symptoms, cosmetic concerns, or documented continued growth were included. Therefore, these findings should not be generalized to all benign thyroid nodules, particularly smaller or asymptomatic ones.

The 2020 European Thyroid Association guideline recommends image-guided thermal ablation as an alternative to surgery for patients with benign thyroid nodules causing pressure symptoms or cosmetic concerns, particularly when patients decline surgery or when surgery may be associated with increased risk [9]. Similarly, the American Thyroid Association statement on ablation techniques emphasizes careful patient selection, confirmation of benignity, appropriate operator expertise, and structured imaging follow-up when thermal ablation is used for benign thyroid nodules [4]. Although these international recommendations do not provide specific age-based indications for thermal ablation, they emphasize individualized decision-making based on clinical symptoms, comorbidities, procedural risk, patient preference, and expected benefit [4,9]. This approach is particularly relevant to older adults, in whom multimorbidity and reduced physiological reserve may increase the risks associated with thyroid surgery. In our study, despite a significantly greater comorbidity burden, older patients achieved volume reduction and clinical improvement comparable to those of younger adults, with no major complications. Thus, our results are consistent with the general principles of current international guidelines and provide additional age-specific evidence suggesting that chronological age alone should not exclude appropriately selected older patients from consideration for MWA.

When examining the demographic characteristics of our study population, women predominated in both the older and younger adult groups, which is consistent with the well-established female predominance of benign thyroid nodules reported in epidemiological studies [3,21]. This sex difference has been attributed to hormonal influences, particularly the effects of estrogen on thyroid cell proliferation and nodule development. Although the prevalence of thyroid nodules increases with advancing age [2,19,20], the proportion of older patients in our cohort was smaller than that of younger adults. This discrepancy most likely reflects the retrospective nature of the study and referral patterns rather than the true epidemiology of thyroid nodules. Older patients are often managed conservatively because of multimorbidity, frailty, increased perceived surgical risk, or patient preference, which may reduce referral for interventional procedures such as MWA. Nevertheless, our findings demonstrate that carefully selected older patients achieved clinical and radiological outcomes comparable to those of younger adults, supporting the feasibility of MWA as a minimally invasive treatment option irrespective of chronological age.

One of the most clinically relevant findings was the similar volume reduction achieved in both age groups. Median volume reduction reached approximately 49–53% at 3 months and 66% at 6 months without significant between-group differences. These findings indicate that tissue response to thermal injury is largely preserved in older patients despite age-related alterations in tissue repair and vascularity. This observation is consistent with previous studies demonstrating that MWA provides progressive and sustained nodule shrinkage over time. Liu et al. reported a mean volume reduction of approximately 90% at 12 months, while Wu et al. similarly observed marked volume regression after one year of follow-up [22,23]. Furthermore, recent meta-analyses have confirmed that MWA achieves substantial and durable volume reduction comparable to other thermal ablation techniques across different patient populations, including older patients [24,25]. Therefore, the current 6-month results most likely represent an intermediate stage of nodule involution rather than the maximal therapeutic response, and additional follow-up would be expected to demonstrate further reduction in nodule volume.

An additional important finding of the present study was the significant improvement in symptom and cosmetic scores following MWA [26]. Although cosmetic concerns are often emphasized in younger patients, compressive symptoms such as dysphagia, neck discomfort, and airway compression may have greater clinical implications in patients because they can adversely affect nutritional status, functional independence, and health-related quality of life [27]. Previous studies have similarly demonstrated that thermal ablation improves symptom burden, cosmetic outcomes, and patient-reported quality of life in patients with benign thyroid nodules [28]. Therefore, the comparable improvement observed in both age groups suggests that MWA provides clinically meaningful benefits beyond radiological volume reduction and further supports the expanding role of minimally invasive treatments in the management of older patients with benign thyroid nodules.

Preservation of thyroid function is one of the major advantages of microwave ablation (MWA), particularly in older patients with multiple comorbidities. In the present study, serum TSH, free T3, and free T4 levels remained stable throughout the 6-month follow-up despite the inclusion of euthyroid, hypothyroid, and hyperthyroid patients. These findings are consistent with previous reports demonstrating that MWA selectively destroys nodular tissue while preserving sufficient surrounding thyroid parenchyma to maintain endocrine function [29,30]. Although experimental and clinical studies have shown that MWA may induce transient biochemical changes immediately after treatment because of thermal injury and the release of preformed thyroid hormones [31], these alterations generally resolve during follow-up and rarely result in permanent thyroid dysfunction. Similarly, Erturk et al. reported transient decreases in TSH and increases in free T3 and free T4 within the first 24 h after MWA, with complete normalization by 6 months [32]. Therefore, the preservation of thyroid function observed in our cohort is consistent with the current evidence and further supports the safety of MWA, particularly in older patients, in whom avoiding treatment-induced hypothyroidism may simplify the management of multimorbidity and polypharmacy. Regarding thyroid functional status, overt hyperthyroidism and antithyroid medication use were excluded, and none of the patients with subclinical hyperthyroidism had an autonomously functioning or toxic nodule. Therefore, the present findings may not be directly applicable to patients with overt hyperthyroidism or toxic thyroid nodules. Future studies that specifically include patients with hyperfunctioning or toxic thyroid nodules may help clarify whether thyroid functional status influences the efficacy and safety of MWA.

Our correlation analyses provided several novel observations. Mixed thyroid nodules demonstrated greater volume reduction than solid or cystic nodules, while increasing aCCI scores were associated with a higher prevalence of mixed nodules. Mixed nodules may respond more favorably because the cystic component collapses rapidly following thermal coagulation and fluid resorption, thereby accelerating overall volume reduction [33]. Furthermore, the association between greater comorbidity burden and mixed nodule morphology may reflect age-related degenerative changes within thyroid tissue rather than a direct causal relationship [34]. Although these findings require confirmation in larger prospective cohorts, they suggest that baseline nodule composition may influence treatment response more strongly than patient age itself. 

Safety is particularly important when considering interventions in older patients. In the present study, no major complications occurred, and only two younger patients experienced transient hoarseness that resolved completely during follow-up. Although the absence of complications in the older cohort should be interpreted cautiously because of the relatively limited sample size, these findings nevertheless support the favorable safety profile of MWA reported in previous studies. Careful hydrodissection, continuous ultrasound guidance, and meticulous protection of surrounding critical structures probably contributed to these excellent procedural outcomes [35].

An important strength of the present study is the direct comparison of older and younger adults treated using a standardized protocol by a single experienced operator, thereby minimizing procedural variability. In addition, incorporation of the age-adjusted Charlson Comorbidity Index provides clinically relevant information regarding the influence of multimorbidity on treatment outcomes, an aspect rarely investigated in previous MWA studies focusing on benign thyroid nodules.

Several limitations should also be acknowledged. First, the retrospective single-center design may introduce selection bias. Second, the relatively modest sample size, particularly in the older subgroup (*n* = 33), limits statistical power for detecting uncommon or rare complications. Therefore, the absence of major complications in our cohort should not be interpreted as evidence that rare adverse events cannot occur. Third, follow-up was limited to six months, whereas maximal nodule shrinkage after MWA frequently continues for 12 months or longer. Finally, thyroid autoantibodies and quality-of-life assessments were not routinely evaluated, preventing more comprehensive assessment of endocrine and patient-reported outcomes. Prospective multicenter studies with larger geriatric cohorts, longer follow-up, and broader inclusion criteria are needed to validate these findings and identify predictors of long-term treatment success. Future research should also evaluate patients with smaller or asymptomatic nodules, as well as those with hyperfunctioning thyroid nodules, to determine the generalizability of MWA outcomes across different clinical presentations.

The duration of follow-up is also important when interpreting both the safety and potential economic implications of MWA. In the present study, follow-up was limited to 6 months, during which no major complications occurred and the two cases of transient hoarseness resolved completely. However, a 6-month observation period may not fully capture delayed complications, long-term regrowth, incomplete ablation, or the need for repeat treatment, all of which could influence the overall effectiveness and cost of MWA over time. From an economic perspective, minimally invasive treatment may potentially reduce costs associated with hospitalization, general anesthesia, surgical complications, postoperative recovery, and long-term thyroid hormone replacement compared with surgery [36]. These potential advantages may be particularly relevant in older patients, in whom postoperative complications and prolonged recovery may generate additional healthcare utilization. Nevertheless, no formal cost-effectiveness or cost-utility analysis was performed in the present study; therefore, any economic advantage of MWA cannot be directly established from our data. Longer-term prospective studies incorporating healthcare resource utilization, repeat interventions, complication-related costs, quality-of-life outcomes, and direct comparisons with surgical management are needed to determine the true cost-effectiveness of MWA in older adults.

## 5. Conclusions

Overall, our findings suggest that microwave ablation offers substantial clinical benefit while maintaining an excellent safety profile in older patients with symptomatic benign thyroid nodules. Given the increasing prevalence of thyroid nodules in ageing populations and the growing burden of multimorbidity, MWA may represent an attractive first-line minimally invasive alternative to surgery for appropriately selected patients.

## Figures and Tables

**Table 1:** Demographic, clinical, and laboratory characteristics of the patients.

	Older Patients (N = 33)	Adult Patients (N = 65)	95% CI	*p*
Age * (±SD)	68.4 ± 6.9	43.7 ± 8.1	(−27.80–−21.50)	<0.001
Sex (FM/M)	26/7	49/16	(−0.14–21.40)	0.7
aCCI			(−5.01–−3.50)	<0.001
Score 0	5 (15.1%)	43 (66.1%)		
Score 1	14 (42.4%)	21 (32.3%)		
Score 2	1 (3%)	1 (1.5%)		
Score 3	2 (3%)	0		
Score 4	8 (24.2%)	0		
Score 5	3 (9.1%)	0		
TSH, Md (IQR)	0.9 [0.4–1.4]	1 [0.6–1.5]	(−1.00–0.32)	0.11
fT3, Md (IQR)	3.9 [3.1–4.6]	3.4 [2.7–3.7]	(−0.97–−0.25)	0.02
fT4, Md (IQR)	1.2 [0.9–1.5]	1.2 [1.1–1.3]	(−1.00–2.80)	0.79

* Since the data were normally distributed. Abbreviations: aCCI, age-adjusted Charlson Comorbidity Index; FM, female; fT3, free triiodothyronine; fT4, free thyroxine; IQR, Interquartile Range; M, male; Md, Median; SD, standard deviation; TSH, thyroid-stimulating hormone. The fT3, fT4 and TSH data of the patients are from the time before the MWA procedure. The significance level is ≤0.05. Effect size: Cohen’s f = 0.6.

**Table 2:** Structural features of thyroid nodules.

	Older Patients (N = 33)	Adult Patients (N = 65)	95% CI	*p*
Nodule volume, mL, Md (IQR)	11.8 [5.7–21.8]	11.2 [6.1–21.2]	(−8.70–5.00)	0.8
Nodule structure, N (%)			(−0.69–11.00)	0.14
Solid	12 (36.4%)	33 (50.8%)		
Cystic	4 (12.1%)	8 (12.3%)		
Mixed	17 (51.5%)	24 (36.9%)		
Nodule echogenicity, N (%)			(−0.40–0.59)	0.08
Hypoechoic	18 (54.5%)	23 (35.3%)		
Isoechoic	11 (33.3%)	28 (43.2%)		
Hyperechoic	4 (12.1%)	14 (21.5%)		
Nodule vascularity, N (%)			(−0.40–0.15)	0.47
Yes	33 (100%)	64 (98.4%)		
No	0	1 (1.6%)		
Thyroid gland functionality, N (%)			(−0.40–0.05)	0.59
Hypothyroidism	3 (9.1%)	4 (6.2%)		
Euthyroidism	19 (57.6.%)	54 (83%)		
Hyperthyroidism	11 (33.3%)	7 (10.8%)		

Abbreviations: IQR, Interquartile Range; Md, Median. The significance level is ≤0.05. Effect size: Cohen’s f = 0.6.

**Table 3:** Nodule volumes after MWA.

	Basal Volume (mL)	3rd Month Control Volume (mL)	6th Month Control Volume (mL)	*p*
Older patients (N = 33) ^a^	11.8 [5.7–21.8]	7.2 [2.8–10.6]	4.3 [1.5–7.5]	<0.001
Adult patients (N = 65) ^b^	11.2 [6.1–21.2]	5.4 [3–9.5]	3.4 [1.6–6.1]	<0.001

Abbreviations: mL, milliliter. ᵃ^,^ᵇ Pairwise comparisons of baseline, 3-month, and 6-month nodule volumes were significant in both groups (all *p* < 0.001; adjusted significance level, *p* ≤ 0.016). The corresponding 95% confidence intervals were 5.1 to 11.4, −4.2 to −1.9, and −15.5 to −7.2 mL for older patients, and 6.0 to 10.0, −3.0 to −1.7, and −12.8 to −8.0 mL for adult patients, respectively. The significance level is ≤0.016. Effect size: Cohen’s f = 0.25.

## Data Availability

The raw data supporting the conclusions of this article will be made available by the authors on request.

## Abbreviations

The following abbreviations are used in this manuscript:

MWA	Microwave ablation
aCCI	Age-adjusted Charlson Comorbidity Index
VRR	Volume reduction rate
TA	Thermal ablation
FNA	Fine-needle aspiration
US	Ultrasound

## References

[1] Liao L.J., Hung S.F., Cheng P.C., Hsu W.L. (2025). Ultrasonography of incidental thyroid nodules: A systematic review and meta-analysis of prevalence. J. Med. Ultrasound.

[2] Meng L., Zhang X. (2026). Prevalence and associated factors of thyroid nodules in a health examination cohort: An ultrasound-based cross-sectional study using electronic health records. Front. Public Health.

[3] Li Y., Teng D., Ba J., Chen B., Du J., He L., Lai X., Teng X., Shi X., Li Y. (2020). Efficacy and safety of long-term universal salt iodization on thyroid disorders: Epidemiological evidence from 31 provinces of mainland China. Thyroid.

[4] Sinclair C.F., Baek J.H., Hands K.E., Hodak S.P., Huber T.C., Hussain I., Lang B.H.-H., Noel J.E., Papaleontiou M., Patel K.N. (2023). General principles for the safe performance, training, and adoption of ablation techniques for benign thyroid nodules: An American Thyroid Association statement. Thyroid.

[5] Kuo T.C., Chen K.Y., Lai C.W., Wang Y.-C., Lin M.-T., Chang C.-H., Wu M.-H. (2024). Comparison of safety, efficacy, and patient satisfaction with thermal ablation versus endoscopic thyroidectomy for benign thyroid nodules in a propensity-matched cohort. Int. J. Surg..

[6] Zhao H.X., Wei Y., Zhao Z.L., Peng L.-L., Li Y., Wu J., Cao S.-L., Yu N., Yu M.-A. (2025). Clinical study on the relationship between the incidence of complications and tumour size after thermal ablation of benign thyroid nodules. Int. J. Hyperth..

[7] Luo X., Kandil E. (2024). Microwave ablation: A technical and clinical comparison to other thermal ablation modalities to treat benign and malignant thyroid nodules. Gland Surg..

[8] Li X.L., Chen Z.T., Jin Y.J., Xu B.-H., Xu Y.-D., Cao Q., Bo X.-W., Wen J.-X., Ji Z.-B., Fan P.-L. (2025). Microwave ablation for benign thyroid nodules with cosmetic problems and related factors for post-ablative complete relief: A two-center retrospective study. Endocrine.

[9] Papini E., Monpeyssen H., Frasoldati A., Hegedus L. (2020). 2020 European Thyroid Association Clinical Practice Guideline for the Use of Image-Guided Ablation in Benign Thyroid Nodules. Eur. Thyroid. J..

[10] Li S., Guo W., Meng Q., Zhu M., Wei H., Ji F., Tan L., Zhang W. (2023). The association between thyroid-stimulating hormone and thyroid nodules, goiter, and thyroid antibody positivity. Front. Endocrinol..

[11] Zhang J., Zhao W., Jin M., Wang W. (2025). Environmental exposures, sleep, physical activity, and risk of thyroid nodules in older adults: A cross-sectional study. Front. Endocrinol..

[12] Charlson M., Szatrowski T.P., Peterson J., Gold J. (1994). Validation of a combined comorbidity index. J. Clin. Epidemiol..

[13] Na D.G., Lee J.H., Jung S.L., Kim J.H., Sung J.Y., Shin J.H., Kim E.-K., Lee J.H., Kim D.W., Park J.S. (2012). Radiofrequency ablation of benign thyroid nodules and recurrent thyroid cancers: Consensus statement and recommendations. Korean J. Radiol..

[14] Tessler F.N., Middleton W.D., Grant E.G., Hoang J.K., Berland L.L., Teefey S.A., Cronan J.J., Beland M.D., Desser T.S., Frates M.C. (2017). ACR Thyroid Imaging, Reporting and Data System (TI-RADS): White Paper of the ACR TI-RADS Committee. J. Am. Coll. Radiol..

[15] Aleman R., Dip F., Rancati A., Rosenthal R.J. (2026). From reaction to prevention: Rethinking vocal fold paralysis after thyroid surgery. Gland Surg..

[16] Fung M.M.H., Lang B.H.H. (2022). Using Intra-Operative Laryngeal Ultrasonography as a Real-Time Tool in Assessing Vocal Cord Function During Radiofrequency Ablation of the Thyroid Gland. World J. Surg..

[17] Bo X.W., Lu F., Xu H.X., Sun L.P., Zhang K. (2020). Thermal ablation of benign thyroid nodules and papillary thyroid microcarcinoma. Front. Oncol..

[18] Bo X.W., Lu F., Yu S.Y., Yue W.-W., Li X.-L., Hu M., Wu L.-L., Lv Z.-Y., Sun L.-P., Xu H.-X. (2022). Comparison of efficacy, safety, and patient satisfaction between thermal ablation, conventional/open thyroidectomy, and endoscopic thyroidectomy for symptomatic benign thyroid nodules. Int. J. Hyperth..

[19] Mammen J.S.R. (2023). Thyroid and aging. Endocrinol. Metab. Clin. N. Am..

[20] Martínez-Montoro J.I., Doulatram-Gamgaram V.K., Olveira G., Valdés S., Fernández-García J.C. (2023). Management of thyroid dysfunction and thyroid nodules in the ageing patient. Eur. J. Intern. Med..

[21] Park S.I., Baek J.H., Lee D.H., Chung S.R., Song D.E., Kim W.G., Kim T.Y., Sung T.-Y., Chung K.-W., Lee J.H. (2024). Radiofrequency ablation for the treatment of benign thyroid nodules: 10-year experience. Thyroid.

[22] Liu Y.J., Qian L.X., Liu D., Zhao J.F. (2017). Ultrasound-guided microwave ablation in the treatment of benign thyroid nodules in 435 patients. Exp. Biol. Med..

[23] Wu W., Gong X., Zhou Q., Chen X., Chen X., Shi B. (2017). US-guided percutaneous microwave ablation for the treatment of benign thyroid nodules. Endocr. J..

[24] Ding J., Wang D., Zhang W., Xu D., Wang W. (2023). Ultrasound-guided radiofrequency and microwave ablation for the management of patients with benign thyroid nodules: Systematic review and meta-analysis. Ultrasound Q..

[25] Qian Y., Li Z., Fan C., Huang Y. (2024). Comparison of ultrasound-guided microwave ablation, laser ablation, and radiofrequency ablation for the treatment of elderly patients with benign thyroid nodules: A meta-analysis. Exp. Gerontol..

[26] Javadov M., Karatay E., Ugurlu M.U. (2021). Clinical and functional results of radiofrequency ablation and microwave ablation in patients with benign thyroid nodules. Saudi Med. J..

[27] Yogaraj V., Sinclair C., Tchernegovski A., Phyland D. (2025). The prevalence of local symptoms in benign thyroid disease: A systematic review with meta-analysis. Laryngoscope.

[28] Kowalski R., Park A., Abdulgader L., Shelawala N., Don R., Ryan K., Johnson A., McArdle P.F., Terhune J., Kuo J.H. (2025). Quality of life following thermal ablation of benign thyroid nodules: A systematic review and meta-analysis. Thyroid.

[29] Heck K., Happel C., Grünwald F., Korkusuz H. (2015). Percutaneous microwave ablation of thyroid nodules: Effects on thyroid function and antibodies. Int. J. Hyperth..

[30] Chen Z., Guo X., Yin X., Wang K., Zhang S., Li J. (2021). Ultrasound-guided microwave ablation of benign thyroid nodules: Effects on inflammatory factors and thyroid function. Am. J. Transl. Res..

[31] Chu K.F., Dupuy D.E. (2014). Thermal ablation of tumours: Biological mechanisms and advances in therapy. Nat. Rev. Cancer.

[32] Erturk M.S., Cekic B., Celik M., Ucar H. (2021). Microwave ablation of symptomatic benign thyroid nodules: Short- and long-term effects on thyroid function tests, thyroglobulin and thyroid autoantibodies. Clin. Endocrinol..

[33] Wang B., Han Z.Y., Yu J., Cheng Z., Liu F., Yu X.L., Chen C., Liu J., Liang P. (2017). Factors related to recurrence of benign non-functioning thyroid nodules after percutaneous microwave ablation. Int. J. Hyperth..

[34] Haugen B.R., Alexander E.K., Bible K.C., Doherty G.M., Mandel S.J., Nikiforov Y.E., Pacini F., Randolph G.W., Sawka A.M., Schlumberger M. (2016). 2015 American Thyroid Association management guidelines for adult patients with thyroid nodules and differentiated thyroid cancer: The American Thyroid Association Guidelines Task Force on Thyroid Nodules and Differentiated Thyroid Cancer. Thyroid.

[35] Cui T., Jin C., Jiao D., Teng D., Sui G. (2019). Safety and efficacy of microwave ablation for benign thyroid nodules and papillary thyroid microcarcinomas: A systematic review and meta-analysis. Eur. J. Radiol..

[36] Yue W.W., Wang S.R., Li X.L., Teng C., Qian L. (2018). Microwave ablation compared to thyroidectomy to treat benign thyroid nodules. Int. J. Hyperth..

